# Assessing the care of doctors, nurses, and nursing technicians for people in situations of sexual violence in Brazil

**DOI:** 10.1371/journal.pone.0249598

**Published:** 2021-11-15

**Authors:** Liene Martha Leal, Maria Auxiliadora Figueredo Vertamatti, Victor Zaia, Caio Parente Barbosa

**Affiliations:** 1 Centro Universitário FMABC, Santo André, São Paulo, Brazil; 2 Universidade Federal do Delta do Parnaíba, Parnaíba, Piauí, Brazil; University of Toronto, CANADA

## Abstract

This study aimed to assess the quality of care for people in situations of sexual violence in health services, identifying positive and negative indicators, and suggest solutions. This is a cross-sectional study with a quantitative approach and convenience sampling. The sample consisted of 134 professionals (doctors, nurses, and nursing technicians) working in public health services. Three instruments were used, namely, a structure evaluation form, a questionnaire, and a process evaluation form. The results revealed eight positive indicators (adequate infrastructure; rooms for patient assistance; gynecological bed; visual and auditory privacy; waiting rooms; a professional team comprising physicians, nurses, nursing technicians, and receptionists; adequate training of staff to provide health services to people in situations of sexual violence; and most healthcare professionals asking their patients about possible sexual violence situations) and nine negative indicators (reduced number of rooms for patient assistance with toilets; absence of protocols to identify and assist people in situations of sexual violence; absence of leaflets, posters, and other materials on sexual violence; absence of a referral flow chart (specific for people in situations of sexual violence) to specialized services; reduced number of consultations with suspected and/or confirmed cases of sexual violence; non-use of specific protocols; not referral of these patients to the specialized care network; most professionals consider the health unit where they work as unable to help people in situations of sexual violence; a decrease in attendance at health facilities that do not have a protocol for assisting people in situations of sexual violence), making clear the interventions necessary to promote the provision of quality health services that meet the specific needs of people in situations of sexual violence. These indicators are expected to provide subsidies for the improvement of public policies aimed at listening, welcoming, identifying, and treating people in situations of sexual violence.

## Introduction

Sexual violence encompasses a variety of behaviors, contacts, and interactions of a non-consensual sexual nature; sexual comments or jokes that cause discomfort; being forced to touch another person’s genitals; be penetrated orally, vaginally, or anally by a penis, or other parts of the body, or objects; being forced to penetrate another person or to practice oral sex with him; be forced to watch or participate in films or photographs and be forced to engage in prostitution, among others [1].

Sexual violence can happen in public and private spaces (home, school, street, workplace), on the internet and can victimize men, women, children, adolescents and the elderly, regardless of race, color, age, religion, education, and socioeconomic level [1, 2]. Each year it is estimated that an average of twelve million people suffers from sexual violence in the world [3]. Sexual violence is a public health problem that results in countless physical, psychological, social, and family health problems, in the short and long term, affecting mainly the sexual and reproductive areas [2, 4, 5].

In Brazil, there is a lot of underreporting in registered cases of sexual violence [3]. It is estimated that in Brazil 90% of cases of sexual violence are not reported [6]. Many of these people seek health services with physical, psychological, or sexual complaints, but do not mention having suffered sexual violence [7]. What demands from professionals working in health services, in addition to the technical-scientific knowledge inherent to their professional practice, specialized listening, aimed at welcoming and identifying patients who are in a situation of sexual violence [8].

According to Donabedian, the evaluation of quality in health services involves the Structure–Process–Outcome triad [9]. Structure refers to physical facilities, equipment, human resources, and flow chart of the health unit’s operation [9]. Process refers to assisting under technical and scientific standards, such as preventive actions, diagnosis, treatment, and patient rehabilitation [9]. Outcome involves professional efficiency, resoluteness, and the satisfaction of the users of the health services [9].

Research conducted in South Africa, Australia, Belgium, Brazil, Canada, El Salvador, United States, Ghana, Guatemala, Honduras, Nicaragua, and Nigeria on the quality of care in health services for people in situations of sexual violence pointed out as positive indicators: humanized treatment; believing the testimony of the victims; receive information about procedures; having the option to choose interventions; adequate staff training; having a mental health service; secrecy and privacy; have the adequate infrastructure; and resoluteness [10].

These surveys revealed as negative indicators: non-humanized care; not believing the testimony of the victims; not receiving information about the procedures; not having the option to choose; lack of adequate staff training; improve mental health services; absence of secrecy and privacy; absence of supplies; ignorance of health professionals about sexual violence in people of the male gender; absence of specific protocols; more clearly define the roles and responsibilities of health professionals and forensic specialists; absence of discipline on sexual violence in undergraduate courses in health; access problems due to lack of information about these services; and access problems due to the stigma and silence surrounding people in situations of sexual violence [10].

This study aimed to assess the quality of care for people in situations of sexual violence in health services, identifying positive indicators that contribute to improving the quality of care for these patients, negative indicators that may decrease the quality of care, and suggest solutions.

## Materials and methods

### Design

This is a cross-sectional study with a quantitative approach, carried out in a public hospital and 33 Basic Health Units in a city in the interior of Brazil in 2018. This study is based on Donabedian’s Structure–Process–Outcome triad to evaluate quality in healthcare [9]. The authors chose not to evaluate the outcome category because of the logistical difficulty of actively seeking people in situations of sexual violence in the health services surveyed, to preserve the privacy of these patients and avoid more distress and possible revictimization [11]. Researches demonstrated that the Donabedian model is not only a validated approach to assess the safety and quality of a health service [12–14], but also serves as a basis for auditing [15].

This project received ethical approval from the Ethics Committee of the Universidade Federal do Piauí - UFPI, under number 60546916.4.0000.5669. Each participant received an Informed Consent Form and was informed about the research objectives, highlighting voluntary participation. The Informed Consent Form was given to the participant in two copies, which were signed by the researcher responsible for data collection and by the participant, who was handed a copy.

### Participants

This study included doctors, nurses, and nursing technicians working in public health services. Participation was voluntary, characterizing a convenience sample (not probabilistic). The professionals answered a questionnaire. The inclusion criterion was to be a professional aged 18 or over. The exclusion criterion was not to answer one or more questions in the questionnaire. The sample consisted of 134 professionals, 23.9% (n = 32) doctors, 25.4% (n = 34) nurses and 50.7% (n = 68) of whom were nursing technicians. A total of 85.1% (n = 114) were female. The average age was 33.0 years (SD = 10.3) and the average length of service was 5.0 years (60.0 months, SD = 103.1). As for education, 50.7% (n = 68) had technical / professional education, 12.7% (n = 17) had an undergraduate degree, 35.8% (n = 48) had a graduate degree, and 0.8% (n = 1) had a master’s degree.

### Instruments

This study used three instruments, namely, a structure evaluation form, a questionnaire, and a process evaluation form. The questionnaire was answered by the professionals who participated in the study and the structure evaluation and process evaluation forms were completed by the researcher responsible for data collection.

Structure Evaluation Form: The form was developed by the authors to allow the evaluation of public healthcare facilities using 14 quality indicators, which were divided into three parameters. The "Physical Resources/Facilities" parameter verified whether there is a waiting room, an assistance room, an office with a toilet, and a gynecological bed and whether there is visual and auditory privacy in the office to be used. The "Human Resources" parameter verified whether there are physicians, nurses, nursing technicians, and receptionists in the unit. The "Protocol and Information Instruments on Sexual Violence" parameter verified whether the unit has written policies and/or procedures to identify and assist people in a situation of sexual violence, leaflets, posters, and other materials on sexual violence, and a referral flow chart to the network of partner services. In this study, the terms “service network” and “assistance network” refer to an articulated set of actions and services from different areas, such as health, social assistance, justice, and public security, whose objective is to identify, assist, and refer people in a situation of sexual violence appropriately [16].

A quality rating system based on Arantes was used to give specific values for each of the three parameters, in which the best quality standard would be 5, and the worst, 0 [17]. Therefore, the quality indicators were scored as follows: 0 for non-existent and 5 for existent. Considering that each parameter had a different quality indicator value, the maximum possible score for each parameter was 30 points in “Physical Resources/Facilities,” 20 in “Human Resources,” and 20 in “Protocol and Information Instruments on Sexual Violence.”

Questionnaire: The professionals answered a questionnaire developed by the authors, containing 12 questions: 1) Professional; 2) Age; 3) Sex; 4) Education; 5) Length of service; 6) Do you ask your patients about possible situations of sexual violence when it is suspected?; 7) Have you ever treated suspected and/or confirmed cases of sexual violence?; 8) Did you use any specific protocol during the care of these patients?; 9) Did you make any referrals during the care of these patients?; 10) Would the health unit you work in would be able to handle cases of sexual violence?; 11) Have you received any training on how to handle sexual violence cases at least once in your life?; 12) What are the main difficulties you face in your work in cases of sexual violence? (S1 Table).

Process Evaluation Form: This is an instrument that was developed by the authors that verify five quality indicators for health professionals; the indicators were divided into two parameters. The "Procedures" parameter verifies whether the professional asks a patient about possible sexual violence situations, whether they have already assisted suspected and/or confirmed sexual violence cases, and whether they used any specific protocol and referred the patient. The "Knowledge on Sexual Violence" parameter verifies whether they received training and qualification on how to assist sexual violence cases at least once in their life. The specific scores for each of the two parameters were determined with the use of a quality rating system based on Arantes, in which the best quality standard was rated 5, and the worst, 0 [17]. Therefore, the quality indicators were scored as follows: 0 for non-existent and 5 for existent. Considering that each parameter had a different value of quality indicators, the maximum possible score in each parameter was 20 points in “Procedures” and 5 points in “Knowledge on Sexual Violence.”

### Data collection procedures

Data were collected individually at the workplace of the professionals who participated in the study. The professionals answered the questionnaire in a self-applicable form and with an average duration of 20 minutes. The public health services were evaluated and scored by the researcher responsible for data collection, according to the structure evaluation form and the questionnaire answered by the professionals were evaluated and scored by the researcher responsible for data collection, according to the process evaluation form.

### Data analysis

To evaluate the structure, after scoring each of the three parameters, each parameter’s mean was calculated by dividing the score obtained by the number of quality indicators for the parameter evaluated, giving each parameter a final score between 0 and 5 points (S2 Table). The final classification of the healthcare unit was expressed as “expected quality standard” (> 4.0 points), “acceptable quality standard” (> 3.0 and < 4.0 points), or “insufficient quality standard” (< 3.0 points). This classification is based on Arantes [17].

To evaluate the process, the data were analyzed as follows: the scores obtained for each of the two parameters by each professional working in the unit were added and then divided by the number of professionals in that unit that participated in the research (mean). Thus, we obtained the final score of the unit in each parameter, which varied from 0 to 5 points (S3 Table). The final classification of the parameters evaluated for the process quality of the unit was the same used to classify the structure.

The independent variables studied were those related to the care for survivors of sexual violence and included: professional (doctor, nurse, and nursing technician), age (<33 years or> 33 years), sex (male and female), education (had technical/professional education, undergraduate degree, graduate degree, and master’s degree), length of service (<5 years or> 5 years), training (yes or no), suitable unit (yes or no) and if the unit where they work has a protocol for assisting sexual violence survivors (yes or no) (S4 Table).

The outcome variables evaluated in this study were: if the professional asks his patients about possible situations of sexual violence (yes or no), if the professional treated suspected and/or confirmed cases of sexual violence (yes or no), if the professional used any specific protocol during the care of these patients (yes or no) and if some referral was made during the care of these patients (yes or no) (S5 Table).

All variables were presented in terms of absolute frequency and percentage. The association between variables was assessed using the Chi-square test, with p-value < 0.05 being considered as statistically significant associations, with a 95% confidence interval. The data were analyzed using R software (The R Project for Statistical Computing) version 3.4.4.

## Results

The structure evaluation showed that 76.5% (n = 26) of the facilities had an acceptable quality standard, 20.6% (n = 7), an insufficient quality standard, and 2.9% (n = 1), an expected quality standard. A health unit, in this case, the state hospital, reached the expected quality standard, achieving excellence in the quality of the structure.

As for the parameters evaluated, 82.4% (n = 28) of the analyzed public health services had an expected quality standard, 14.7% (n = 5), an acceptable quality standard, and 2.9% (n = 1), an insufficient quality standard in “Physical Resources/Facilities.” As for “Human Resources,” 94.1% (n = 32) obtained an expected quality standard, and 5.9% (n = 2), an acceptable quality standard. However, 97.1% (n = 33) obtained an insufficient quality standard, and 2.9% (n = 1), an acceptable quality standard in the “Protocol and Information Instruments on Sexual Violence” parameter.

The quality indicators evaluated in the "Physical Resources/Facilities" parameter showed that 100% (n = 34) of the studied healthcare facilities had a room for patient assistance; 82.4% (n = 28) had a gynecological table; 97.1% (n = 33) had a waiting room and visual and auditory privacy. However, 73.5% (n = 25) did not have an office with a toilet.

As for the quality indicators evaluated in the “Human Resources” parameter, 100% (n = 34) of the analyzed public health facilities had nurses, nursing technicians, and receptionists; 94.1% (n = 32) of them had a doctor.

As for the quality indicators evaluated in the "Protocol and Information Instruments on Sexual Violence" parameter, 100% (n = 34) of the analyzed public health facilities had no written policies and/or procedures (a protocol) to identify people in situations of sexual violence; 97.0% (n = 33) had no written policies and/or procedures (a protocol) to assist people in situations of sexual violence; 64.7% (n = 22) had no leaflets, posters, and other materials on sexual violence; and 100% (n = 34) had no referral flow chart (specific for people in situations of sexual violence) to the network of partner facilities.

The process evaluation showed that 91.2% (n = 31) of the public health facilities had an insufficient quality standard, 5.9% (n = 2), an expected quality standard, and 2.9% (n = 1), an acceptable quality standard. Two health units, the state hospital, and primary healthcare units obtained the expected quality standard, achieving excellence in the quality of the process.

According to the parameters evaluated, 88.2% (n = 30) of the public health facilities had an insufficient quality standard, 5.9% (n = 2), an acceptable quality standard, and 5.9% (n = 2), an expected quality standard in “Procedures.” As for “Knowledge on Sexual Violence,” 38.2% (n = 13) obtained an insufficient quality standard, 32.4% (n = 11), an acceptable quality standard, and 29.4% (n = 10), an expected quality standard.

According to the quality indicators evaluated in the “Procedures” parameter, 62.7% (n = 84) of health professionals asked their patients about possible sexual violence situations; 14.9% (n = 20) assisted suspected and/or confirmed sexual violence cases. During these consultations, 70.0% (n = 14) of these professionals used no specific protocol and 80.0% (n = 16) referred these patients. As for the quality indicators evaluated in the “Knowledge on Sexual Violence” parameter, 87.3% of health professionals received training and qualification on how to assist sexual violence cases.

The data analysis showed that 53.7% (n = 72) of the professionals consider the healthcare unit where they work as not able to assist people in situations of sexual violence. These professionals reported the following as among the main difficulties they face in their work with sexual violence cases: lack of training and qualification (37.3%, n = 50), lack of communication with the specialized assistance network (13.4%, n = 18), a people in situations of sexual violence fear of talking about the violence suffered (12.7%, n = 17), knowing how to approach the people in situations of sexual violence (10.5%, n = 14), lack of follow-up to the victim and the family (8.2%, n = 11), lack of protocol (6.7%, n = 9), lack of flowchart (6.0%, n = 8), the resistance of the family (3.7%, n = 5), and lack of structure (1.5%, n = 2).

There was an association, with statistical significance (p = 0.032), between education and referral. The most educated professionals were the ones who most referred the survivors of sexual violence to the specialized assistance network (Table 1).

**Table 1:** Associations between forwarding and independent variables.

Independent variables	Some referral was made during the care of these patients				p-value
	Yes		No		
	n	%	n	%	
Professional					
Doctor	3	18.8	0	0.0	0.468^1^
Nurse	6	37.5	1	25.0	
Nursing technician	7	43.8	3	75.0	
Age					
≤ 33 years	8	50.0	4	100.0	0.068^1^
> 33 years	8	50.0	0	0.0	
Sex					
Male	2	12.5	1	25.0	0.531^1^
Female	14	87.5	3	75.0	
Education					
Had technical/professional education	7	43.8	3	75.0	0.032^1^*
Undergraduate degree	0	0.0	1	25.0	
Graduate degree	9	56.3	0	0.0	
Length of service					
≤ 5 years	6	37.5	1	25.0	0.639^1^
> 5 years	10	62.5	3	75.0	
Training					
Yes	14	87.5	4	100.0	0.456^1^
No	2	12.5	0	0.0	
Suitable unit					
Yes	9	56.3	2	50.0	0.822^1^
No	7	43.8	2	50.0	
Unit has protocol					
Yes	1	6.3	1	25.0	0.264^1^
No	15	93.8	3	75.0	

^1^Chi-square test

*significance probability value (p <0.05).

n = number of individuals varies due to the characteristics of the variable.

There was an association, with statistical significance (p = 0.016), between care for survivors of sexual violence and the absence of protocols in health units. There is a decrease in attendance at health facilities that do not have a protocol for assisting survivors of sexual violence (Table 2).

**Table 2:** Associations between care for survivors of sexual violence and independent variables.

Independent variables	The professional treated suspected and/or confirmed cases of sexual violence				p-value
	Yes		No		
	n	%	n	%	
Professional					
Doctor	3	15.0	4	7.0	0.352^1^
Nurse	7	35.0	15	26.3	
Nursing technician	10	50.0	38	66.7	
Age					
≤ 33 years	12	60.0	28	49.1	0.402^1^
> 33 years	8	40.0	29	50.9	
Sex					
Male	3	15.0	8	14.0	0.916^1^
Female	17	85.0	49	86.0	
Education					
Had technical/professional education	10	50.0	33	57.9	0.568^1^
Undergraduate degree	1	5.0	6	10.5	
Graduate degree	9	45.0	17	29.8	
Length of service					
≤ 5 years	7	35.0	33	57.9	0.07^1^
> 5 years	13	65.0	24	42.1	
Training					
Yes	18	90.0	49	86.0	0.644^1^
No	2	10.0	8	14.0	
Suitable unit					
Yes	11	55.0	25	43.9	0.390^1^
No	9	45.0	32	56.1	
Unit has protocol					
Yes	2	10.0	0	0.0	0.016^1^*
No	18	90.0	57	100.0	

^1^Chi-square test

*significance probability value (p <0.05).

n = number of individuals varies due to the characteristics of the variable.

In the structure evaluation, the health units analyzed presented an acceptable quality standard. In the process evaluation, the quality standard was considered insufficient. This means that the health facilities evaluated have the physical structure and human resources necessary to care for people in situations of sexual violence, however, changes are necessary for the care processes for these patients. As shown in Table 3, results obtained in the three instruments applied revealed eight positive indicators and nine negative indicators, making clear the interventions necessary to promote the provision of quality health services that meet the specific needs of people in situations of sexual violence.

**Table 3:** Positive and negative indicators resulting from the evaluation of the quality of care provided to people in situations of sexual violence in health services.

Positive indicators	Negative indicators
Adequate infrastructure	Reduced number of rooms for patient assistance with toilets
Rooms for patient assistance	Absence of protocols to identify and assist people in situations of sexual violence
Gynecological bed	Absence of leaflets, posters, and other materials on sexual violence
Visual and auditory privacy	Absence of a referral flow chart (specific for people in situations of sexual violence) to specialized services
Waiting rooms	Reduced number of consultations with suspected and/or confirmed cases of sexual violence
A professional team comprising physicians, nurses, nursing technicians, and receptionists	Non-use of specific protocols
Adequate training of staff to provide health services to people in situations of sexual violence	Not referral of these patients to the specialized care network
Most healthcare professionals asking their patients about possible sexual violence situations	Most professionals consider the health unit where they work as unable to help people in situations of sexual violence
	A decrease in attendance at health facilities that do not have a protocol for assisting people in situations of sexual violence

## Discussion

The performance of health professionals is extremely important for making an early diagnosis, for the intervention and prevention of problems resulting from sexual violence [8]. Researchers defend the inclusion of assistance to people in situations of sexual violence in the undergraduate curriculum of medical and nursing students [2, 18–20].

The main difficulties pointed out by professionals in cases of sexual violence were lack of training, lack of communication with the specialized service network, and lack of knowledge on how to approach the patient. Studies carried out in Belgium, Ghana, and Kenya on the evaluation of care for people in situations of sexual violence in health services revealed the need for additional training for health professionals involved in the care of these patients, more specifically for the care of children [21–24]. It is recommended to draw up an annual training agenda with the aim of training and sensitizing professionals to identify and meet the specific needs of each case. The themes worked on would include the definition of sexual violence, the myths, and beliefs about sexual violence, the specificities of differences in gender, age, and sexual orientation, the epidemiology of sexual violence, the legislation regarding the assistance to people in situations of sexual violence, the physical, psychological, social and family problems resulting from sexual violence, emergency contraception, sexually transmitted diseases, interventions, and necessary laboratory and forensic exams, and the importance of humanized care and welcoming and responsible listening.

The absence of clinical protocols and specific guidelines for the treatment of people in situations of sexual violence, especially children and adolescents, also compromises the quality of care for these patients [18, 23, 25, 26]. It is recommended to develop a protocol for the identification of patients in situations of sexual violence and another protocol for the care of this population, defining which professional will be responsible for each stage of care (initial interview, history record, clinical examination and gynecological, forensic examinations, psychological monitoring, medication and notification to Organs competent bodies).

There is a great stigma surrounding sexual violence that will be reflected in the silence of people in situations of sexual violence [22, 23]. Most of the survivors remain silent, keeping their suffering to themselves. This silence is perpetuated by the family, by the community and will be reflected in the silence of the health professional who does not listen to the reception and identification of patients in situations of sexual violence, which generates a great underreporting of registered cases, compromising adequate care for the recovery of the physical and mental health of these patients [3, 22, 27]. It is necessary to identify the hidden, the unspoken, in the speech of people in situations of sexual violence who seek health services. The presence of leaflets, posters, and other materials with information on sexual violence in healthcare facilities would also contribute to breaking this silence. The lack of information material on sexual violence corroborates studies conducted in Nigeria [25] and Brazil [2, 18].

A limitation of this study was convenience sampling, which makes generalizations impossible. However, our results corroborate those of previous studies, demonstrating the importance of evaluating the quality of health care for people in situations of sexual violence.

Sexual violence requires an interdisciplinary practice, covering three main axes: health, social assistance, and justice. Further research is suggested involving the evaluation of care for people in situations of sexual violence in social assistance and justice services.

## Conclusions

The quality of care for people in situations of sexual violence in health services includes an adequate infrastructure; patient care rooms with bathrooms; gynecological bed; visual and auditory privacy; waiting rooms; professional team composed of doctors, nurses, nursing technicians, and receptionists; adequate training of staff to provide health services to people in situations of sexual violence; health professionals questioning their patients about possible situations of sexual violence; protocols to identify and assist people in situations of sexual violence; leaflets and a referral flowchart (specific for people in situations of sexual violence) for specialized services. The presence of leaflets, posters, and informational material on sexual violence in health facilities would help reduce the silence of people in situations of sexual violence and would also contribute to the improvement of care for these patients. It is hoped that the indicators raised here can contribute to the improvement of public policies aimed at listening, welcoming, identifying, and treating people in situations of sexual violence.

## Supporting information

S1 Table Questionnaire. (DOCX)

S2 Table Structure evaluation form. (DOCX)

S3 Table Process evaluation form. (DOCX)

S4 Table Distribution of independent variables. (DOCX)

S5 Table Structure evaluation form. (DOCX)
