# Supplementary material for: Assessing the care of doctors, nurses, and nursing technicians for people in situations of sexual violence in Brazil
Source: PLoS One. 2021 Nov 15;16(11):e0249598. doi: 10.1371/journal.pone.0249598 (PMC8592427; doi:10.1371/journal.pone.0249598)
Supplement: S1 Table — (DOCX) [file pone.0249598.s001.docx]

| **S1 Table. Questionnaire.** |
| --- |
| **Questions** |
| 1. Professional: ( ) Doctor ( ) Nurse ( ) Nursing Technician  2. Age:  3. Sex: ( ) Male ( ) Female  4. Education:  ( ) Technical / professional education. ( ) Undergraduate degree.  ( ) Graduate degree. ( ) Master’s degree.  5. Length of service:  6. Do you ask your patients about possible situations of sexual violence when it is suspected?  ( ) No ( ) Yes  7. Have you ever treated of suspected and / or confirmed cases of sexual violence?  ( ) No ( ) Yes  8. Did you use any specific protocol during the care of these patients?  ( ) No ( ) Yes.  9. Did you make any referrals during the care of these patients?  ( ) No ( ) Yes.  10. Would the health unit you work in would be able to handle cases of sexual violence?  ( ) No ( ) Yes  11. Have you received any training on how to handle sexual violence cases at least once in your life?  ( ) No ( ) Yes  12. What are the main difficulties you face in your work in cases of sexual violence? |
