# Supplementary material for: Assessing the care of doctors, nurses, and nursing technicians for people in situations of sexual violence in Brazil
Source: PLoS One. 2021 Nov 15;16(11):e0249598. doi: 10.1371/journal.pone.0249598 (PMC8592427; doi:10.1371/journal.pone.0249598)
Supplement: S2 Table — (DOCX) [file pone.0249598.s002.docx]

| **S2 Table.** **Structure evaluation form.** | | | | | | |
| --- | --- | --- | --- | --- | --- | --- |
| **INSTITUTION:** | | | | | | |
| **PARAMETER** **1)** **PHYSICAL RESOURCES/FACILITIES** | | | | | | |
| QUALITY INDICATORS | | | SCORE | | | |
| Is there a waiting room? | | | ( ) Existent (5 points) | | | ( ) Non-existent (0 point) |
| Is there at least one answering room? | | | ( ) Existent (5 points) | | | ( ) Non-existent (0 point) |
| Is there at least one an doctor's office with a toilet? | | | ( ) Existent (5 points) | | | ( ) Non-existent (0 point) |
| In the office that will be used, can patients not be seen from the outside? | | | ( ) Existent (5 points) | | | ( ) Non-existent (0 point) |
| In the office that will be used, can not the conversations be heard from outside? | | | ( ) Existent (5 points) | | | ( ) Non-existent (0 point) |
| Is there gynecological stretcher in the room where the care will be performed? | | | ( ) Existent (5 points) | | | ( ) Non-existent (0 point) |
| TOTAL OF POINTS | | |  | | | |
| AVERAGE (Points collected divided by 06) | | |  | | | |
| **PARAMETER** **2) HUMAN RESOURCES** | | | | | | |
| QUALITY INDICATORS | | | SCORE | | | |
| Is there a doctor in the unit? | | | ( ) Existent (5 points) | | | ( ) Non-existent (0 point) |
| Is there a nurse in the unit? | | | ( ) Existent (5 points) | | | ( ) Non-existent (0 point) |
| Are there nursing staff in the unit? | | | ( ) Existent (5 points) | | | ( ) Non-existent (0 point) |
| Is there a receptionist in the unit? | | | ( ) Existent (5 points) | | | ( ) Non-existent (0 point) |
| TOTAL OF POINTS | | |  | | | |
| AVERAGE (Points collected divided by 04) | | |  | | | |
| **PARAMETER** **3) PROTOCOL AND INFORMATION INSTRUMENTS ON SEXUAL VIOLENCE** | | | | | | |
| QUALITY INDICATORS | | | SCORE | | | |
| Is there in the unit written policies and / or procedures (a protocol) to **identify** persons in situations of sexual violence? | | | ( ) Existent (5 points) | | | ( ) Non-existent (0 point) |
| Is there in the unit written policies and / or procedures (a protocol) for **care** of the person in situations of sexual violence? | | | ( ) Existent (5 points) | | | ( ) Non-existent (0 point) |
| Are there pamphlets, posters, and other material on sexual violence in the unit? | | | ( ) Existent (5 points) | | | ( ) Non-existent (0 point) |
| Is there a flowchart in the unit for referrals to the partner services network? | | | ( ) Existent (5 points) | | | ( ) Non-existent (0 point) |
| TOTAL OF POINTS | | |  | | | |
| AVERAGE (Points collected divided by 04) | | |  | | | |
| PARAMETERS | | POINTS GOT | | AVERAGE INDICATORS | | |
| 1) PHYSICAL RESOURCES/FACILITIES - 06 quality indicators. | |  | | (Points collected divided by 06) | | |
| 2) HUMAN RESOURCES - 04 quality indicators. | |  | | (Points collected divided by 04) | | |
| 3 PROTOCOL AND INFORMATION INSTRUMENTS ON SEXUAL VIOLENCE - 04 quality indicators. | |  | | (Points collected divided by 04) | | |
| **FINAL SCORE (Sum of the means of the indicators divided by 03)** | | | |  | | |
| **FINAL CLASSIFICATION** |  | | | | | |
| FINAL CLASSIFICATION - Parameter 1 |  | | | | | |
| FINAL CLASSIFICATION - Parameter 2 |  | | | | | |
| FINAL CLASSIFICATION - Parameter 3 |  | | | | | |
| FINAL CLASSIFICATION | CRITERIA | | | | VALUES | |
| EXPECTED QUALITY STANDARD | 80% or more | | | | > 4.0 points | |
| ACCEPTABLE QUALITY STANDARD | between 60 and 79% | | | | > 3.0 and <4.0 points | |
| INSUFFICIENT QUALITY STANDARD | less than 60% | | | | <3.0 points | |
