# Supplementary material for: Assessing the care of doctors, nurses, and nursing technicians for people in situations of sexual violence in Brazil
Source: PLoS One. 2021 Nov 15;16(11):e0249598. doi: 10.1371/journal.pone.0249598 (PMC8592427; doi:10.1371/journal.pone.0249598)
Supplement: S3 Table — (DOCX) [file pone.0249598.s003.docx]

| **S3 Table.** **Process evaluation form.** | | | | | | |
| --- | --- | --- | --- | --- | --- | --- |
| **INSTITUTION:** | | | | | | |
| **ASSESSED PROFESSIONAL:** | | | | | | |
| **PARAMETER** **1) PROCEDURES** | | | | | | |
| QUALITY INDICATORS | | SCORE | | | | |
| Do you question patients about possible sexual violence? | | ( ) Existent (5 points) | | | | ( ) Non-existent (0 point) |
| Did you handle suspected and / or confirmed cases of sexual violence? | | ( ) Existent (5 points) | | | | ( ) Non-existent (0 point) |
| Did you use any specific protocol during these cases? | | ( ) Existent (5 points) | | | | ( ) Non-existent (0 point) |
| Did you make any referrals during the care of these patients? | | ( ) Existent (5 points) | | | | ( ) Non-existent (0 point) |
| TOTAL OF POINTS | |  | | | | |
| AVERAGE (Points collected divided by 04) | |  | | | | |
| **PARAMETER** **2) KNOWLEDGE ON SEXUAL VIOLENCE** | | | | | | |
| QUALITY INDICATORS | | SCORE | | | | |
| Have you received any training on how to handle sexual violence cases at least once in your life? | | ( ) Existent (5 points) | | | | ( ) Non-existent (0 point) |
| TOTAL OF POINTS | |  | | | | |
| AVERAGE (Points collected divided by 02) | |  | | | | |
| **PARAMETER** **1 - ASSESSED PROFESSIONALS** | | | | AVERAGE OBTAINED | | |
| Doctor | | | |  | | |
| Nurse | | | |  | | |
| Nursing Technician | | | |  | | |
| Nursing Technician | | | |  | | |
| TOTAL (sum of the averages obtained by the evaluated professionals) | | | |  | | |
| FINAL AVERAGE (sum of the averages obtained by the evaluated professionals divided by the number of evaluated professionals) | | | |  | | |
| **PARAMETER** **2 - ASSESSED PROFESSIONALS** | | | | AVERAGE OBTAINED | | |
| Doctor | | | |  | | |
| Nurse | | | |  | | |
| Nursing Technician | | | |  | | |
| Nursing Technician | | | |  | | |
| TOTAL (sum of the averages obtained by the evaluated professionals) | | | |  | | |
| FINAL AVERAGE (sum of the averages obtained by the evaluated professionals divided by the number of evaluated professionals) | | | |  | | |
| PARAMETERS | | | FINAL AVERAGE OBTAINED | | | |
| 1) PROCEDURES | | |  | | | |
| 2) KNOWLEDGE OF SEXUAL VIOLENCE | | |  | | | |
| **FINAL SCORE (Sum of the** **final** **averages** **obtained in the two dimensions divided by 02)** | | |  | | | |
| **FINAL CLASSIFICATION** |  | | | | | |
| FINAL CLASSIFICATION - Parameter 1 |  | | | | | |
| FINAL CLASSIFICATION - Parameter 2 |  | | | | | |
| FINAL CLASSIFICATION | CRITERIA | | | | VALUES | |
| EXPECTED QUALITY STANDARD | 80% or more | | | | > 4.0 points | |
| ACCEPTABLE QUALITY STANDARD | between 60 and 79% | | | | > 3.0 and <4.0 points | |
| INSUFFICIENT QUALITY STANDARD | less than 60% | | | | <3.0 points | |
