# Supplementary material for: Assessing the care of doctors, nurses, and nursing technicians for people in situations of sexual violence in Brazil
Source: PLoS One. 2021 Nov 15;16(11):e0249598. doi: 10.1371/journal.pone.0249598 (PMC8592427; doi:10.1371/journal.pone.0249598)
Supplement: S4 Table — (DOCX) [file pone.0249598.s004.docx]

| **S4 Table. Distribution of independent variables.** | | |
| --- | --- | --- |
| Independent variables | n | % |
| Professional |  |  |
| Doctor | 32 | 23.9 |
| Nurse | 34 | 25.4 |
| Nursing technician | 68 | 50.7 |
| Age |  |  |
| < 33 anos | 69 | 51.5 |
| > 33 anos | 65 | 48.5 |
| Sex |  |  |
| Male | 20 | 14.9 |
| Female | 114 | 85.1 |
| Education |  |  |
| Had technical / professional education | 68 | 50.7 |
| Undergraduate degree | 17 | 12.7 |
| Graduate degree | 48 | 35.8 |
| Master's degree | 1 | 0.8 |
| Length of service |  |  |
| < 5 anos | 40 | 51,9 |
| > 5 anos | 37 | 48,1 |
| Training |  |  |
| Yes | 117 | 87.3 |
| No | 17 | 12.7 |
| Suitable unit |  |  |
| Yes | 62 | 46.3 |
| No | 72 | 53.7 |
| Unit has protocol |  |  |
| Yes | 4 | 3.0 |
| No | 130 | 97.0 |
