# Supplementary material for: Assessing the care of doctors, nurses, and nursing technicians for people in situations of sexual violence in Brazil
Source: PLoS One. 2021 Nov 15;16(11):e0249598. doi: 10.1371/journal.pone.0249598 (PMC8592427; doi:10.1371/journal.pone.0249598)
Supplement: S5 Table — (DOCX) [file pone.0249598.s005.docx]

| **S5 Table. Distribution of outcome variables.** | | |
| --- | --- | --- |
| Outcome variables | n | % |
| The professional asks his patients about possible situations of sexual violence |  |  |
| Yes | 84 | 62.7 |
| No | 50 | 37.3 |
| The professional treated suspected and / or confirmed cases of sexual violence |  |  |
| Yes | 20 | 14.9 |
| No | 114 | 85.1 |
| The professional used any specific protocol during the care of these patients |  |  |
| Yes | 6 | 30.0 |
| No | 14 | 70.0 |
| Some referral was made during the care of these patients |  |  |
| Yes | 16 | 80.0 |
| No | 4 | 20.0 |
